# In vitro interaction of colloidal nanoparticles with mammalian cells: What have we learned thus far?

**DOI:** 10.3762/bjnano.5.161

**Published:** 2014-09-09

**Authors:** Moritz Nazarenus, Qian Zhang, Mahmoud G Soliman, Pablo del Pino, Beatriz Pelaz, Susana Carregal-Romero, Joanna Rejman, Barbara Rothen-Rutishauser, Martin J D Clift, Reinhard Zellner, G Ulrich Nienhaus, James B Delehanty, Igor L Medintz, Wolfgang J Parak

**Affiliations:** 1 Fachbereich Physik, Philipps-Universität Marburg, Renthof 7, 35037 Marburg, Germany; 2 CIC Biomagune, Paseo Miramón 182, 20009 San Sebastian, Spain; 3 BioNanomaterials, Adolphe Merkle Institute, University of Fribourg, Route de L’ancienne Papeterie CP 209, Marly 1, 1723, Fribourg, Switzerland; 4 Institute of Physical Chemistry, University of Duisburg-Essen, Universitätsstraße 5, 45141 Essen, Germany; 5 Institute of Applied Physics and Institute of Toxicology and Genetics, Karlsruhe Institute of Technology (KIT), Wolfgang-Gaede-Straße 1, 76131 Karlsruhe, Germany; 6 Department of Physics, University of Illinois at Urbana-Champaign, 1110 West Green Street, Urbana, IL 61801, USA; 7 Center for Bio/Molecular Science & Engineering, Code 6900, U.S. Naval Research Laboratory, 4555 Overlook Avenue Southwest, Washington D.C., 20375, USA

**Keywords:** colloidal stability, intracellular particle distribution, nanoparticles, protein corona, toxicity of nanoparticles

## Abstract

The interfacing of colloidal nanoparticles with mammalian cells is now well into its second decade. In this review our goal is to highlight the more generally accepted concepts that we have gleaned from nearly twenty years of research. While details of these complex interactions strongly depend, amongst others, upon the specific properties of the nanoparticles used, the cell type, and their environmental conditions, a number of fundamental principles exist, which are outlined in this review.

## Introduction

**Figure 1:**
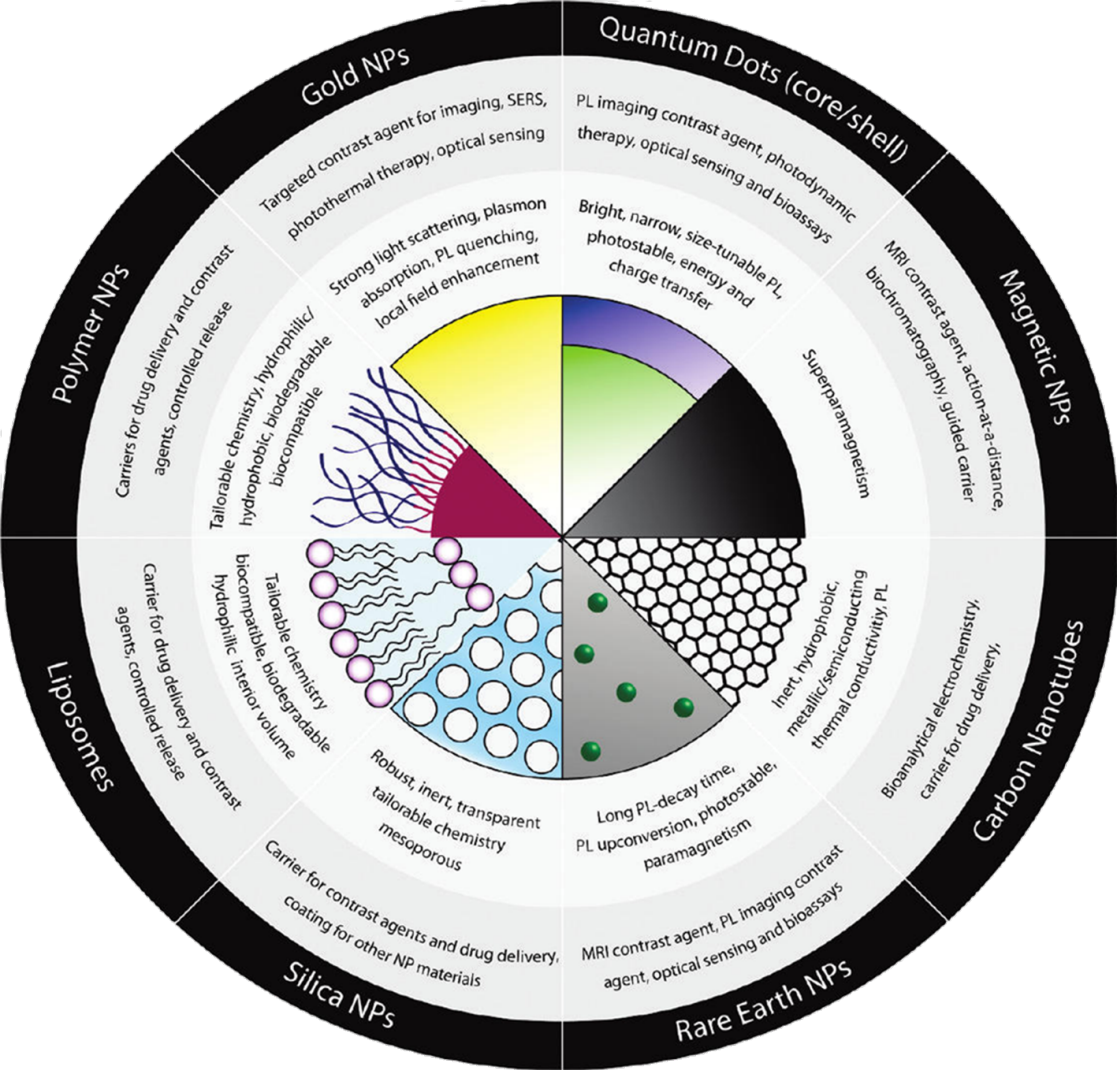
An overview of the “zoo” of different NPs concerning their composition, functionality, and fields of application. Reproduced with permission from [11]. Copyright (2011) American Chemical Society.

**Figure 2:**
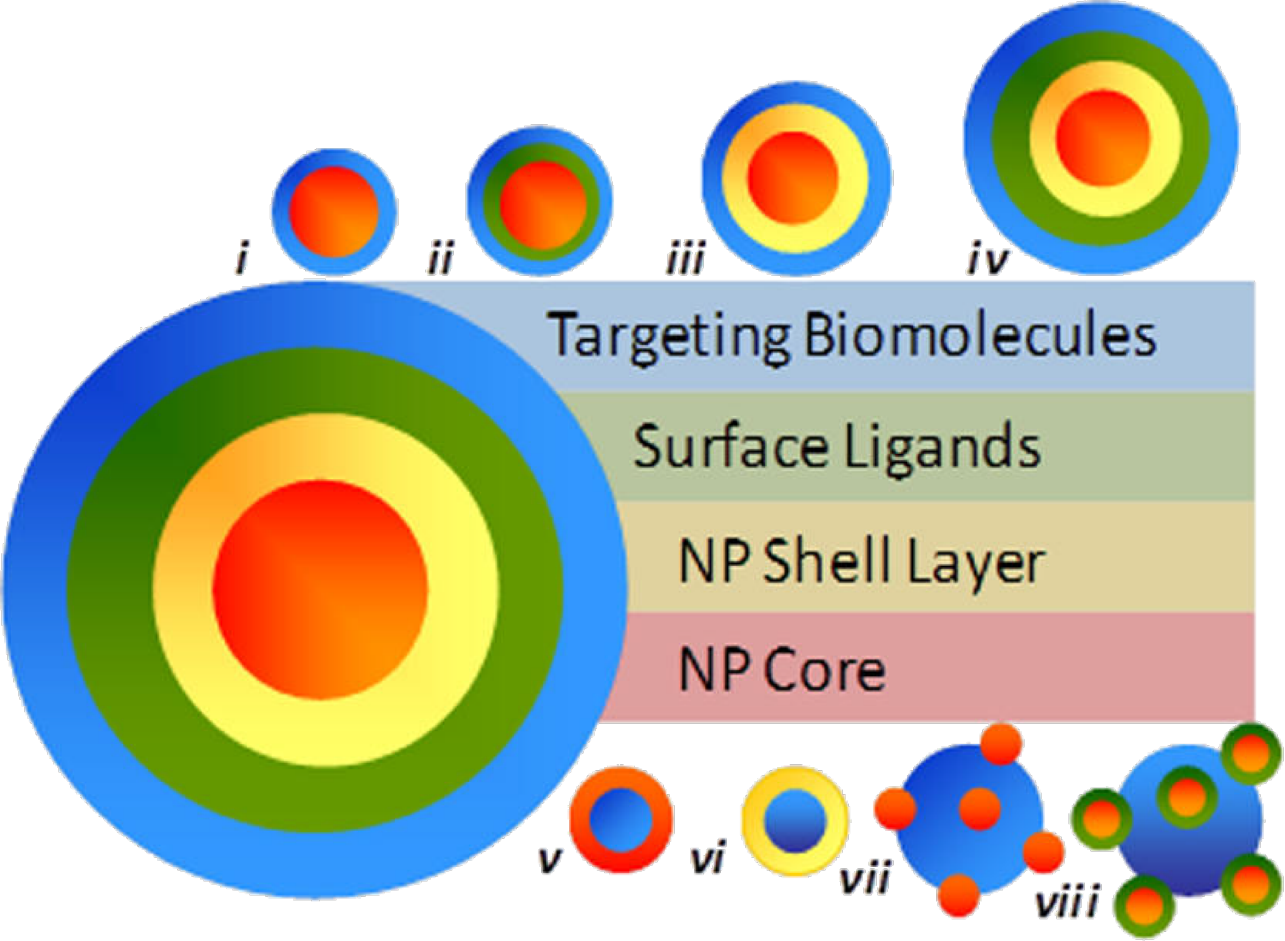
Hybrid nature of typical NPs, comprising different structural compartments. Reproduced with permission from [13]. Copyright (2013) American Chemical Society.

There is a multitude of reports about the interaction of colloidal nanoparticles (NPs) with mammalian cells [1], as this topic is important for analyzing intended (e.g., medical applications [2–4]) and non-intended (e.g., contamination [5–7]) exposure of NPs to humans. However, there is a great number of available NPs made of many different materials [8–10] with a wide range of different functionalities, cf. Figure 1. For a classification of NPs according to their composition, functionality, and fields of application we refer to a recent review [11]. To complicate the situation, most NPs do not consist of only one substance, but typically are hybrid materials, involving surface coatings and other modifications [12], cf. Figure 2 [13]. Even a homogeneous NP formed out of only one material will turn effectively into a hybrid NP, when it is brought into contact with any biological system (e.g., biological media) because of an organic coating that will form on the surface of the NPs [14]. This all illustrates that virtually no two types of NPs are the same and their inherent structure, properties, and constituent materials will contribute to the way in which they are taken up by cells. For example, a 20 nm diameter polymeric dendrimer may be very flexible, whereas a 20 nm metal NP may not, which leads to different interaction with cells. Furthermore, all of these different NPs can be exposed to different cells (e.g., macrophages, endothelia, and tumor cells) under different exposure scenarios (in vitro and in vivo), which as a consequence culminates in a large, but diverse body of work reported in the literature [15–17]. Due to this overwhelming amount of data, it is not easy to obtain a comprehensive overview. Many studies focus on the details of particular systems, but those can dramatically vary from case to case, and even conflicting trends are reported [17]. In addition, results will depend on the cellular test model used. In order to simplify the discussion, this review focuses on in vitro interaction of NPs with adherent, mammalian, immortalized cell lines. This avoids for example the problem of having to discuss how NPs reach and penetrate tissue, which makes in vivo scenarios more complicated than in vitro test systems. Despite these issues, it is still possible to discern some general trends, as described within this review. However, a limitation to having general trends equates to being permissive of some specific details, though common agreements reported here are clearly not trivial. It also automatically involves the possibility that studies exist, which under particular experimental conditions claim the opposite to the general statements. The most important of these trends will be discussed. In this regard, the current review will focus on physicochemically defined NPs, i.e., solutions of monodisperse NPs with a defined ligand shell attached, and without residual “left-over” impurities of the NP synthesis [13,18].

## Review

### How do particles enter cells and where do they go?

**Figure 3:**
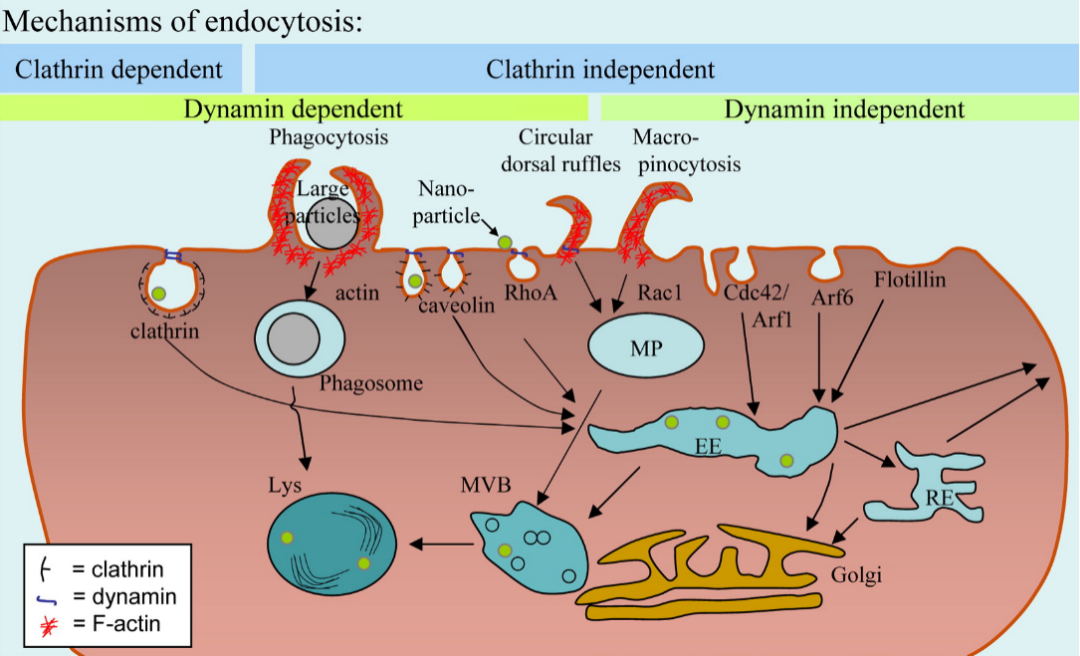
Scheme depicting the different mechanisms of cellular endocytosis. Reproduced with permission from [41]. Copyright (2011) Elsevier.

**Figure 4:**
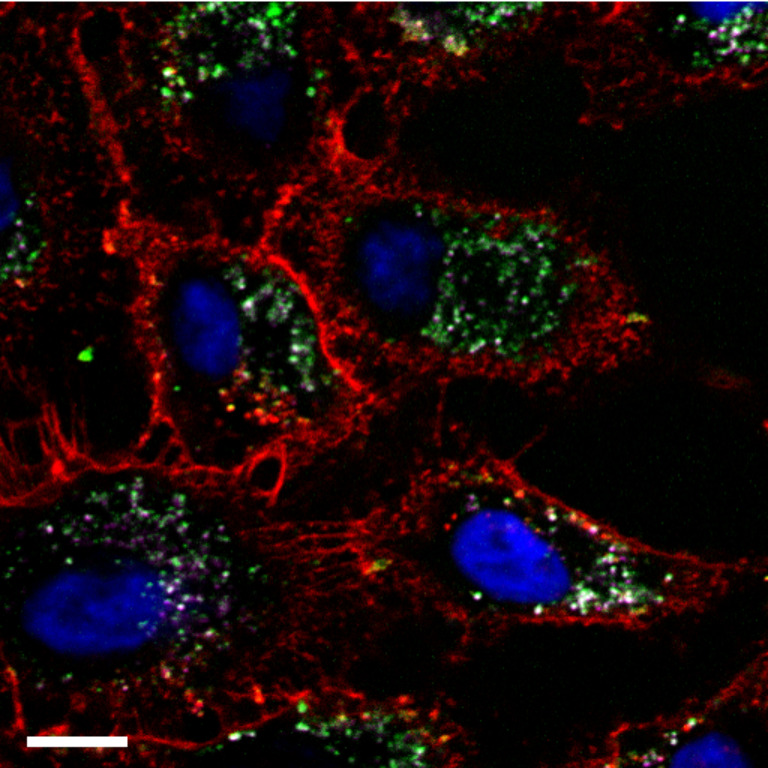
Fluorescence microscopy image showing the granular structure of internalized NPs inside A549 lung cancer cells (two types of iron oxide NPs with different surface chemistry, labelled with different fluorophores (green and magenta)) after 24 h of incubation at a concentration of 1 µg/mL, which are located in individual vesicles. Nuclei are stained with DAPI (blue) and the cell membrane with Wheat Germ Agglutinin (red). Note that due to limited lateral resolution of optical microscopy the spots most likely do not correspond to individual NPs, but to several NPs, which are entrapped inside intracellular vesicles. The scale bar represents 5 μm. Adopted with permission from [65] und Creative Commons Attribution 4.0 International Public License.

**Figure 5:**
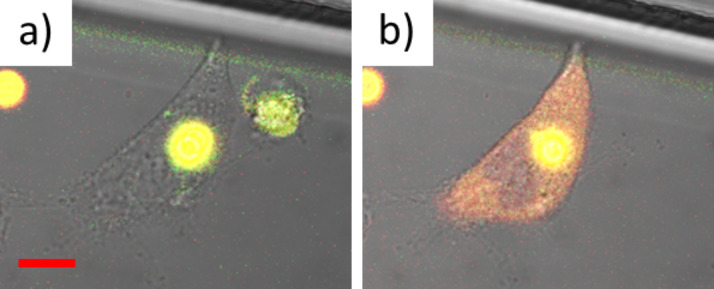
a) A microparticle has been internalized by an A549 lung cancer cell into an intracellular vesicle (here the lysosome [165]) and is thus clearly localized. The microparticle is filled with a pH-sensitive fluorophore (SNARF, from Invitrogen, now LifeTech) linked to dextran and the acidic pH of the lysosome is reported by the yellow fluorescence. b) After release of the pH-sensitive fluorophore linked to dextran to the cytosol (by photothermal heating), the fluorophore–dextran conjugates are freely dispersed, without any visible granular structure. Due to the neutral pH in the cytosol the fluorescence of the fluorophore–dextran conjugates has changed to red. The scale bar corresponds to 10 μm. Adopted with permission from [166]. Copyright (2012) Elsevier.

**Figure 6:**
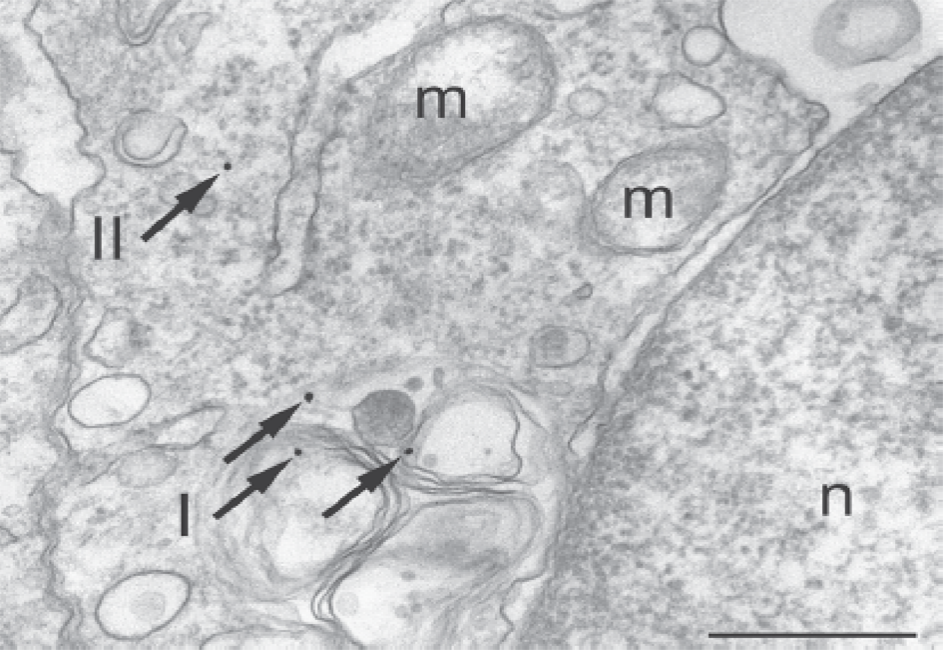
Intracellular compartments after internalization of PEG-coated gold NPs as visualized with TEM. The NPs (which are individually resolved due to the high lateral resolution of TEM) are located within a lysosome (arrows I) and in the cytosol (arrow II). *m* and *n* demark the nucleus and mitochondria, respectively. The scale bar corresponds to 500 nm. Adopted from with permission from [68]. Copyright 2010 Wiley-VCH Verlag GmbH & Co. KGaA, Weinheim.

Virtually all cell lines internalize NPs, which are dispersed in the growth medium [19]. Uptake of different NPs by different cell lines, however, can vary significantly in biological kinetics [20–22] (this is also true for larger microparticles [23]). This is particularly important to keep in mind for specific (i.e., targeted) NP uptake, in which NPs modified by ligands (such as folic acid), which bind to appropriate receptors on the cell surface (such as folate receptors [24]), are specifically internalized [25]. Ligand-mediated uptake (which depends also on the ligand “valence”, i.e., the number of ligands per NP, their density, and their orientation [26]) is faster and more efficient than non-specific (i.e., not receptor-mediated) uptake [27–28], although also plain or non-targeted NPs will be incorporated by cells. Thus, an important parameter to compare amongst studies, in which specific uptake is reported, is the time scale used within the experimental approach. While after short times of exposure huge differences in the amount of incorporated NPs can exist (e.g., between ligand-modified and plain NPs), those differences typically become less significant after longer exposure times [29], e.g., by the presence of the protein corona [30], as will be discussed later in more detail. Thus, statements which claim that only specifically modified NPs, but not non-modified NPs are taken up by cells, have to be regarded highly critically and put into the correct context of the reported time-scale. In fact, differences in uptake are not digital (i.e., "yes" or "no"), but rather are based on different kinetics. However, non-adhesive cell lines, i.e., cell suspensions, can be different and examples in which no significant internalization of NPs happened are reported [31]. Coming back to adhesive cell-lines, the first step in NP internalization obviously is the contact of the NP with the cell plasma membrane. This is a concentration-dependent process, which for high NP concentrations no longer scales linearly with concentration (i.e., saturation effects may occur). The first association of a non-targeted NP with a cell surface is usually electrostatic. Positively charged NPs are, for example, believed to interact with surface-displayed heparan sulfate proteoglycans [32–33]. As a rule of thumb, NPs which strongly interact with the cell plasma membrane, be it by ligand–receptor-mediated or by charge-mediated adhesion, are also internalized more efficiently [34]. Non-fouling polyethylene glycol (PEG)-modified NPs, for example, stick less to the cell plasma membrane and are, therefore, incorporated by cells less efficiently than other NPs [35–37] (this is also true in vivo as manifested by enhanced retention times [38]). It is clear that, while there are a number of portals through which NPs can gain entry into the cell, they all have as the common denominator the cell plasma membrane. Thus, the NP either must translocate (diffuse) directly across the cell plasma membrane entering the cytosol, or it must be internalized via any of the several routes of cellular endocytosis. While some evidence exists to support the direct membrane translocation of a select number of NP materials (typically partly hydrophobic and very small, as discussed later) the overwhelming evidence to date supports endocytosis as the common route of NP uptake. Thus, once NPs are associated to the outer cell plasma membrane they are typically internalized by endocytosis [39–40]. While a variety of different endocytotic pathways exist, which can be quite different in detail (to appreciate the complexity of endocytosis, we refer the reader to the review by Iversen et al. [41], cf. Figure 3), all of them have in common that the NPs are surrounded by membrane. Pinching-off of the membrane-surrounded NPs from the cell plasma membrane leaves the NPs incorporated into intracellular vesicles. These vesicles undergo a cascade of intracellular trafficking steps passing the NPs to more and more acidic vesicles [42–43], which also comprise enzymes specialized in digesting nutrition (and thus also parts of the NPs are digested in the lysosome [44–45]). In other words, after incorporation, the majority of NPs is not “free” in the cytosol, but inside intracellular vesicles (cf. Figure 4). Inside those intracellular vesicles the NPs are in an environment (acidic pH, enzymes) completely different from that in the cytosol (cf. Figure 5). Endocytosis and the endosomal escape dilemma have to be taken into account in particular concerning the delivery applications of NPs, in which the goal is to deliver something to the cytosol. Getting stuck inside intracellular vesicles is redundant to the purpose of these applications. However, in contrast to endocytosis as described so far, studies exist in which it is claimed that NPs can directly translocate through the cell membrane, thus indicating alternative pathways for NPs to penetrate the cell plasma membrane [46–47]. Besides other possible mechanisms, passive diffusion through (transient) membrane pores and passive uptake by van der Waals or steric interactions (subsumed as adhesive interactions) have been suggested [48]. Still, it is always important to interpret such studies critically [49]. Most of the time studies involve an analysis of intracellular NP distributions, i.e., they rely on images showing NPs distributed in the cytosol. Additionally, these studies often rely on the observation that cellular NP entry still occurs below physiological temperatures (e.g., 4 °C), at which endocytosis and the active transport machinery are abrogated. However, without probing also for vesicular membranes around the NPs it is complicated to claim that the NPs in fact have passed the cell plasma membrane as "naked" NPs, without having ever been inside any intracellular vesicle. Clearly, there are a lot of indications (e.g., simulations) that NPs can enter cells through transient pore formation, in particular very small NPs [50–51]. Still, in many publications experiments do not unequivocally demonstrate this pathway, though it surely exists. One possibility of experimental modification would involve, for example, pH-sensitive fluorophores (such as SNARF [52–53]) attached to the surface of the NPs, which can distinguish between the neutral cytosol and highly acidic intracellular vesicles [54]. In a similar direction the reductive capacity of glutathione (the cytosolic concentration of which is between 5 and 10 mM) may be used to displace a fluorescence resonance energy transfer (FRET) acceptor on the surface of the NP as confirmation of a successful NP localization to the cytosol [55]. Such experiments are in particular important for distinguishing between direct translocation to the cytosol versus endocytotic uptake followed by endosomal release. In fact, while there is clear experimental proof that NPs can be transported to the cytosol, the most straightforward pathway is uptake through endocytosis followed by release from the intracellular vesicles to the cytosol [56–58] (and not the diffusion through (transient) membrane pores). Endosomal release is, for example, a scenario which has been unraveled in detail for NPs coated with certain cell penetrating peptides (CPPs) [59–62]. Thus, while NPs can be free in the cytosol, this clearly does not automatically involve that they are membrane-permeable and not endocytosed. As pointed out above, observations based on merely measuring intracellular NP distributions are not sufficient for making profound statements about the uptake pathway. On the other hand it is safe to say that different intracellular locations for NPs exist. NPs have been reported in different intracellular organelles such as mitochondria, the nucleus, and free in the cytosol [63–64]. Most of the time such intracellular distributions are analyzed with transmission electron microscopy (TEM), in which also the structure of the intracellular organelles can be resolved (cf. Figure 6), or with fluorescence microscopy, in which the intracellular organelles have been co-stained with a fluorescent marker [65–67]. However, these data have to be interpreted carefully. In particular, such data should always include a quantitative distribution analysis, which is highly time-consuming. Even plain NPs without any particular surface capping can be found free in the cytosol [68], however, only to a very low extent. Thus, images in which NPs are shown in some particular intracellular organelles are only of limited value if the fraction of NPs that resides in these organelles is not quantified. Quantification, however, is not as trivial as it seems, and there is a need for better quantitative techniques for the future. While TEM offers the lateral resolution to visualize individual NPs, typically only a limited amount of cell sections (i.e., thin slices cut from cells) can be observed and thus for an absolute quantification, which is highly time-consuming, stereological tools need to be employed [68–69]. Also in case of TEM studies knowledge and understanding of cells under TEM conditions is essential. Fluorescence, on the other hand, can be recorded quantitatively by assuming that the emission intensity is proportional to the number of NPs. However, fluorescence can be partly quenched in certain organelles (for example at low pH), and it is impossible to resolve individual NPs due to the limited lateral resolution of optical microscopy [70]. In addition, as mentioned before, NPs can be partly degraded after having been internalized [71–72] and thus, in case fluorescently labeled NPs are used, it is required to prove that the fluorescence (or any other) label is still attached to the NPs inside the cells. Otherwise the recorded intracellular distribution of fluorescence may originate from detached labels and thus would not reflect the distribution of the NPs [73]. Summarizing available data suggests that, while translocation from intracellular compartments to the cytosol and from there to other cellular organelles is possible, translocation efficiencies still are moderate at best. In addition, NPs free in the cytosol may later end up again in intracellular vesicles through auto-phagocytosis [74]. Thus, for many applications, such as intracellular sensing or drug delivery, translocation of NPs to the cytosol after spontaneous endocytotic uptake remains a major challenge. External stimuli may be helpful in this direction [75]. In order to close this section it is also important to think about what happens after endocytotic uptake. It is, for instance, often overlooked that there is an eventual loss of the total NP load per cell as a result of mitotic division, NP exocytosis, and NP transcytosis [76]. This is largely due to the fact that in most experimental systems the primary issues addressed are uptake efficiency of the NPs and subsequent intracellular fate. These parameters are typically asked over the time course required for NP internalization and subcellular localization, and are not tracked over long time courses. It is generally accepted that NPs are partitioned during cell division, in which they are passed to the daughter cells [76–77]. Such dilution effect of NP labels is in particular important for studies involving NPs as long-term tracers. Here, the relevant question arises whether upon cell division NPs are passed 50/50 to each daughter cell. Summers et al. have done both a theoretical [78] and experimental assessment [79] showing that, while partitioning of endosomes to daughter cells is symmetric, the number of NPs per endosome is a distribution and therefore NP partitioning to daughter cells is asymmetric. Thus, after several division cycles the NP distribution will not necessarily be representative for the fate of the original “mother” cells anymore. NPs also can be excreted to the extracellular medium, which represents an additional source of NP dilution effects. While endocytosis of NPs has been investigated heavily there are only a limited number of reports investigating exocytosis of NPs [80–82]. Excretion of NPs in exosomes (i.e., membrane surrounded vesicles), however, clearly affects the long-term cellular loading with NPs. In addition, for some particular cells, transcytosis has also been reported, i.e., that NPs are passed from one cell to another one [83].

### What are the critical parameters involved in in vitro nanoparticle internalization?

**Figure 7:**
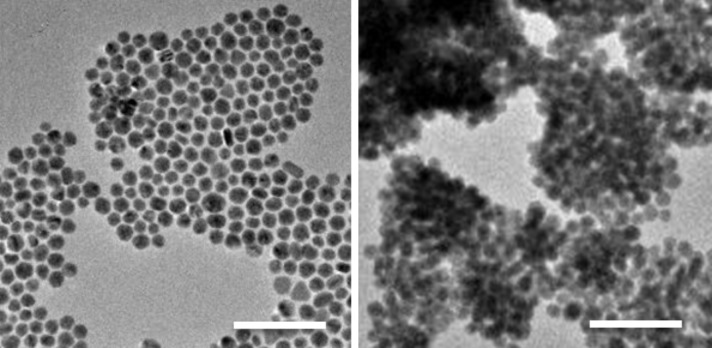
TEM images of a) dispersed and b) agglomerated Au NPs. The scale bars correspond to 100 nm. Adopted with permission from [30]. Copyright 2014 Royal Society of Chemistry.

As mentioned before, virtually all NPs are spontaneously internalized by adherent cells, mainly cell lines, that are usually grown on a certain support and covered with cell culture medium under static conditions. In this case, NPs in the medium can directly access cells, and issues like tissue penetration, which need to be considered in in vivo experiments, can be neglected. The kinetics of internalization can depend strongly on the physicochemical properties of the NPs, the type of cells, and other parameters. Cellular uptake studies of NPs require as much characterization of the NP materials as currently possible. Concerning the NPs, this is, unfortunately, hampered by our incapability to synthesize “defined” NPs. For quantitative studies NPs and their bioconjugates should be as monodisperse as possible with regard to all relevant parameters, such as charge and size, well-defined and well-characterized. Moreover, in the case of bioconjugates, the biological molecule, be it protein or drug, should be attached to the NP with control over orientation [84–85], density, affinity, and number or ratio per NP [85]. Although these goals are extremely hard to achieve, the more they can be fulfilled, the less heterogeneity is present in the NP material and the easier the results (i.e., the correlation between the properties of the NPs and the observed interaction of NPs with cells) can be interpreted [13,18,86]. With limitations, a correlation of the spontaneous endocytotic uptake of NPs to the physicochemical properties of the NPs can be found. One, however, has to be aware that many physicochemical properties of NPs, such as size, shape, charge, and colloidal stability are highly entangled [14]. The physicochemical properties are not intrinsically associated with the NPs, but result from the interaction of the NPs with the surrounding particular medium [87]. The colloidal stability is presumably the most influential parameter. NPs with low colloidal stability will agglomerate and thus, originally "small" NPs will transform into agglomerates, resulting in large particles presented to the cells (cf. Figure 7). However, “colloidal stability” is not a defined physicochemical entity such as size, but needs to be put in context with the measurement protocol, such as the tendency to agglomerate. Any correlation to the size of the NPs without any previous demonstration of colloidal stability in the incubation medium has to be seen very critically. Loss of colloidal stability during incubation also complicates dosimetry. If NPs are quantified in numbers, is an agglomerate of NPs considered to be one particle or the number of NPs in the agglomerate [14]? Agglomeration can have direct consequences on cellular uptake [62]. If the cell cultures are turned upside-down, i.e., the cells are hanging in the culture medium, NP agglomerates that have precipitated at the bottom would not reach the cells and thus the effective NP concentration would be dramatically reduced [88]. In contrast, in conventional geometry, in which the culture medium is on top of the cells, a reduced colloidal stability leads to the precipitation of NP agglomerates onto the cells and, thus, to enhanced uptake, which can influence the cell viability negatively [89]. Such different exposure scenarios are highly relevant for the prediction of NP interactions, for instance, in the human body or in ecotoxicology. Some NPs have been mistaken to elicit limited to no adverse effects upon zebrafish assays, as they had precipitated to the bottom, and thus, the fish had not been directly exposed to them. After correct solubilization, however, the same NPs turned out to be highly detrimental to zebrafish health [90]. Colloidal stability does not only interfere with size but also with other parameters such as shape. An agglomerated bundle of sharp NPs may no longer be "sharp". Thus, colloidal stability is the paramount parameter to consider for all correlations between the NP–cell interactions and the physicochemical properties of the NPs. Reports, in which no characterization of colloidal properties has been performed, therefore have to be regarded very critically. Unfortunately, many NPs are not colloidally stable in cell culture media [91]. The reason is that many NPs are stabilized by charge (in contrast to stabilization through steric repulsion). Salt (in particular NaCl, which always is present at high concentration) in the media screens the NP charge and thus can cause agglomeration [92]. Consequently, data which demonstrate that NPs are colloidally stable in water do not provide any proof that the same NPs also will be stable in cell culture media. Besides salt, proteins are another key compound of (serum-containing) cell media. As discussed later in more detail, proteins adsorb to the surface of NPs, forming the so-called protein corona [93–94], which in fact can increase or reduce colloidal stability [95–96]. Thus, characterization of colloidal stability and other physicochemical properties of NPs needs to be carried out under the same conditions under which later on cells are incubated with the NPs (i.e., in the respective cell culture media [86,97–98]). Obviously, NPs also should be appropriately purified [99], as otherwise effects from impurities rather than from the NPs themselves cannot be excluded. Unfortunately, for unstable NPs (e.g., for NPs to which the organic surface capping is only loosely attached) purification can trigger a loss of colloidal stability and thus agglomeration [14]. As lack of colloidal stability can overrule the other parameters, the following discussion about dependencies of other parameters is done assuming colloidally stable NPs. Uptake of NPs into the cells clearly depends on the size of the NPS. In general, smaller NPs are incorporated by cells faster than bigger ones, though there is some kind of size limit, i.e., the trend does not continue down to ultrasmall NPs [40,100]. As mentioned, upon endocytosis NPs are first wrapped by cellular membrane. Due to intrinsic stiffness and other parameters for membrane bending the radii of curvature cannot become infinitely small, and thus, there is an optimal NP size [101–102]. Excluding ultra-small NPs (smaller than 2–3 nm), smaller NP (smaller than 20–25 nm) are internalized readily in endosomes with most rapid kinetics [103]. Larger NPs (smaller than 60–70 nm) are internalized with lower kinetics to the extent that they are largely associated to the cell membrane over the time courses that see an intake of smaller NPS [62]. This has also been shown in fixed, permeabilized cells (to eliminate cell uptake machinery and pathways) to directly assess the size restrictions of plasma and intracellular membrane barriers on NP passage [104]. In contrast, ultrasmall NPs may be small enough to become membrane-permeable and thus bypass endocytotic uptake. Size-dependent uptake has also been reported for in vivo scenarios [105]. However, in particular for statements concerning size-dependent internalization, the experimental size determination of NP is important. Unfortunately, this is not a straightforward task, as different techniques measure different types of sizes. TEM only provides the geometric size of the NP core which has sufficient contrast, but organic surface cappings are typically not included [14]. In solution there is adsorption of counter ions to the NP surface [106–107] and organic surface coatings can swell, which results in hydrodynamic diameters larger than the core diameters as determined with TEM. There are several techniques for determining the hydrodynamic diameters of NPs [108], of which dynamic light scattering (DLS) might be the most common approach. All techniques have their limitations, and it is always helpful to know the measuring principle they are based on. DLS, for example, is based on calculating autocorrelation functions of the light-scattering signal of the solution. In order to obtain quantitative values, these autocorrelation data need to be fitted with a model, which is, for example, often done by assuming free diffusion of three NP species of different size. Thus, the results are based on the model (which is hidden as "black-box" in the software). To give an example, in case three species are assumed one always will obtain three peaks in the size distribution spectra, even though the sample may contain more different NP species. From the model, diffusion coefficients are yielded as fit parameters, which can be converted to hydrodynamic diameters by the Stokes–Einstein relation. As NPs of larger size also scatter light much more than smaller NPs, the results for DLS-derived size distributions also are quite different depending on whether number or intensity distributions are reported. Thus, simply taking the mean hydrodynamic diameter as displayed by commercial set-ups is prone to errors [109]. Calibration standards of NPs of known size are always a good help to benchmark size measurements and it is highly beneficial to apply several techniques in parallel [108–110]. By applying existing techniques correctly, the hydrodynamic diameters of NPs can be determined with remarkable accuracy, in particular if relative size changes are determined. Detection can be sensitive enough to resolve size-changes due to the attachment of individual macromolecules to the NPs [84–85,111–112]. Besides size, also shape has been proven to modulate the NP uptake of cells. In general, elongated, sharp NPs (i.e., NPs with a prolate spheroid shape) enter cells better than flatter NPs (i.e., NPs with an oblate spheroid shape). This however does no longer hold for very long fibers with high aspect ratios [100]. Flattening of NPs has been used, for example, to reduce NP uptake by cells in a way that flat NPs just adhere to the plasma cell membrane like a “backpack”, without being internalized, in contrast to spherical NPs that are readily incorporated [113–114]. Concerning a third parameter, charged NPs usually are internalized more efficiently than neutral ones, presumably due to enhanced charge-mediated adhesion to the outer cell membrane. Note that the charge pattern of the plasma cell membrane is patchy, and thus, while the overall net charge of cells is negative, there are plenty of positively charged domains. However, due to the overall negative net charge, positively charged NPs are typically incorporated more efficiently by cells than negatively charged ones [65,97,115–119]. Indeed, the current consensus is that positive charges on NPs, such as those provided by the TAT peptide or surface functionalization, interact initially with the negatively charged heparan sulfate proteoglycan groups on the exterior of the cells. This allows them to then be present on the plasma cell membrane as endocytosis starts. Thus, while details may be very complex, clearly some tendencies for which physicochemical parameters enhance the spontaneous endocytosis of NPs can be given. In general, small, elongated, and positively charged NPs are incorporated preferentially to big, flat, and uncharged NPs. Dependency on other physicochemical parameters such as stiffness [120] has not been investigated extensively yet.

### The role of the protein corona

In serum-containing media or inside cells all different types of biologically relevant molecules adsorb to the surface of NPs. i) Ions such as H^+^, Na^+^, K^+^ or Ca^2+^ in the case of negatively charged NPs, or Cl^−^ in the case of positively charged NPs adsorb to the NPs. As a consequence of counter ion adsorption the local ion concentration around the NPs surface is different from the bulk [54,87,106–107]. ii) Also nucleic acids, such as mRNA or siRNA, which are negatively charged due to their phosphate groups attach to positively charged NPs [121–122]. iii) Lipids present in membranes or second-messenger lipids wrap around NPs driven by hydrophilic/hydrophobic interaction and often result in formation of micelles [123–124]. iv) Thiols, present in glutathione or reduced proteins bind to the surface of noble metal NPs, in particular to Au NPs [125–126]. v) Proteins, in general, tend to adsorb to surfaces, which is also true on the nanometer scale. Adsorption of albumin is, for example, an integral part of opsonization [127–128]. The proteins adsorbed to the surface of NPs are typically termed protein corona [93–94]. The protein corona has a significant impact on how NPs interact with cells and thus will be discussed in the following in more detail. NPs can, in principle, be synthesized in water without any organic surface coating, for example by laser ablation [129–131]. However, also to NPs just stabilized by their surface charge (which can be directly on the inorganic surface) proteins will adsorb in serum-containing cell media and in this way can provide additional colloidal stability [129]. Therefore, there are no "naked" NPs in serum-containing cell culture media and inorganic NP cores are always surrounded by an organic coating [14]. Adsorbed proteins can significantly alter the surface properties of NPs and are of key importance in defining the biological identity of NPs [132–133]. The corona formed around NPs is what the cell will “see” primarily, though certainly also the original properties of the underlying NPs determine interactions with the cells [97]. In general, adsorbed proteins "smear out" differences in the surface chemistry between different NPs. Thus, typically two different types of NPs show more pronounced differences in their interaction with cells in case exposure is done in serum-free media (i.e., without proteins) rather than in serum-containing media [97]. The effect of ligands immobilized on the surface of NPs designated for ligand–receptor-mediated uptake is diminished by the protein corona, which partly overcoats the ligands [134]. However, due to the fact that specific targeting still is possible [84], enough ligands still are biologically active. For highly defined NPs, such as nearly monodisperse NPs overcoated with a shell of an amphiphilic polymer [135], the corona formed by special model proteins can be surprisingly well organized. By using fluorescence correlation spectroscopy (FCS), Röcker et al. investigated the adsorption of human serum albumin onto FePt NPs and found clear evidence that the proteins formed a monolayer on the surface of the NP [136]. Additional FCS studies by using other important serum proteins invariably confirmed the formation of monolayer. The thickness of the monolayer could be related to the molecular dimensions of the adsorbed protein determined by X-ray diffraction. All proteins studied were found to adsorb in a specific orientation determined by local charge distributions on the protein surface [20,137–138]. However, adsorption of proteins to the surface of NPs is not only driven by the basic physicochemical properties of the NP such as size, shape, surface charge, but also by other parameters such as the incubation temperature [139]. While model systems involving only one type of NPs and one type of protein help to analytically quantify protein adsorption, such as by determining binding constants [30,136], the biological reality is more complex. Serum-containing cell culture media comprise hundreds of different proteins. To make it worse to analyze, protein adsorption is also a dynamic process. Thus, proteins which are initially bound to the NP surface can later be replaced by others [140–141], which also is referred to as the Vroman effect [142]. It has been shown, for example, that surfactant lipids bound on multiwall carbon nanotubes are replaced with blood plasma proteins after a subsequent incubation [143]. Mass spectrometry is an invaluable tool for quantifying the amounts of different adsorbed protein species [140–141]. The dynamic exchange of proteins, induced by their different adsorption kinetics and affinities to the NP surface is reflected in the discrimination between "soft" and "hard" corona [144–145]. The initial, soft corona is formed by the most abundant proteins, which are then replaced by the high-affinity proteins to yield the hard corona. It has been suggested that differences for different protein species can be characterized by their dissociation constants [30]. In a simple model the dissociation constant tells which protein concentration is required to saturate half of the NP surface with proteins under equilibrium conditions [30]. With simple treatments such as the Hill Model [146] one may characterize the protein corona around NPs with only a few parameters, which would be a great help in comparing results obtained with different systems, thus allowing for a more comprehensive understanding. While the protein corona around NPs has been heavily investigated these data ultimately are only relevant for the first interaction of NPs with cells. After spontaneous endocytosis NPs are inside intracellular vesicles. This imposes a completely different environment than that of the extracellular medium, in particular low pH, presence of endo-/lysosomal enzymes, and different reducing agents [147]. Thus, after NP uptake the protein corona around NPs may change significantly. The original proteins can be displaced by other intracellular proteins, and even more severe, part of the original protein corona may be digested enzymatically [44–45,148–149]. Changes of the protein corona in turn may also alter the physicochemical properties (such as colloidal stability) of the NPs [96]. In this manner, for a full understanding of NP interaction with cells along the pathway of NP uptake the physicochemical characterization of NPs should also be done intracellularly, which, however, is complicated. This opens up a window for future research efforts.

### Toxic effects of NPs

NPs clearly can trigger toxic effects in cells such as cytotoxicity, oxidative stress, (pro-)inflammation, and genotoxicity [150–152]. While again the detailed mechanisms are very complex and by far not understood in a comprehensive way, yet again there are certain characteristic features [153]. Toxic effects can result from the NPs themselves (e.g., by their catalytic surface or by their organic coating, such as in the case of cetyltrimethylammonium bromide (CTAB), a surfactant commonly used to synthesize gold nanorods) or by ions released from the NPs [154–155]. Ion release from certain materials such as Ag, ZnO, or CdSe is in particular triggered by the highly acidic pH in endo-/lysosomal compartments [156]. In both cases adverse biological effects are typically correlated with the production of reactive oxygen species (ROS) [157–158]. Also membrane damage plays a decisive role. In case of dissolvable NPs, the extent to which toxicity originates from the NPs themselves and to which extent from released ions is still subject to an intense scientific debate. Unfortunately, it is experimentally complicated to separate both effects. Even if before exposure all free ions were removed from the NP solution, inside cells new ions would be released. Thus, it is virtually impossible to have cells exposed exclusively to NPs without free ions [87]. One may argue that on the other hand cells could be exposed just to the free ions. While this is true, exposure to free ions will result in different intracellular ion distributions than the one obtained by ions which have been released from the NPs intracellularly, which again complicates direct comparison. Physicochemical properties can be, in some way, correlated with NP toxicity. In other words, reporting toxicity without accompanying in-depth NP characterization is not very useful concerning a detailed understanding of the mechanism. Surface coatings and impurities in the NPs can play an important role. Thus, also the coatings alone, as well as potential impurities need to be investigated towards potential toxic effect in control experiments. If only the physicochemical properties of “pure” NPs are considered, NPs with low colloidal stability have bigger effective sizes, thus are internalized to a larger extent, and thus typically have a greater adverse biological impact [154]. In order to account for concentration effects, it is advisable to correlate toxicity with particle internalization by using adequate methods. Enhanced uptake is one major reason (amongst others) why positively charged NPs (which are incorporated to a higher extent) elicit an increased adverse cellular effect compared to negatively charged ones [97,117–118]. This opens a dilemma. While in general, positive charge is better for enhanced uptake, too much positive charge becomes so toxic that it outweighs the added benefit of enhanced uptake. Thus, for delivery applications an optimum between both effects has to be found. This opens up another important point about the biological impact of NPs that merits discussion. There is a big difference between the use of NPs for cellular labeling or biosensing studies in research, as opposed to any therapeutic (in vivo) utility, and the two should never be thought of together or directly compared. It was, for example, recently shown that semiconductor quantum dot NPs (QDs) were unable to elicit a more negative biological effect when used for cellular labeling than a panel of dyes commonly used for the same intrinsic purposes [159]. Along with this, often transformed and immortalized cell lines are used in biological research, meaning that they are essentially cancerous. Thus, what appears to be adverse biological impact in these experiments has to be qualified with this context in mind. For cellular labeling, perhaps, there is the need for a particular experiment that should drive the issue of toxicity. If the use is specifically for in vitro labeling, tracking or sensing, there are multiple studies that have shown that over the time course required to perform such studies, the impact on cellular viability/proliferation at appropriate NP concentrations is minimal and is often comparable to or even less impactful than the use of traditional materials designed for the same purpose [159]. In this case “chronic toxicity” does not play a role, as the experiment is terminated before such an effect may occur. In contrast, for in vivo delivery one has to consider that NPs will remain in the organism over extended periods of time [160], and thus, benefits of treatment have to be weighted with long-term toxic effects [161]. Consequently, toxicity of NPs always has to be seen in the context of the applications the NPs are used for, but furthermore, the potential accidental exposure beyond the application has to be considered and its risk has to be assessed. In the context of this review we have focused on in vitro studies. The advantage of such studies is the easy screening capability and the possibility to monitor in detail biomolecular pathways and changes in gene expression as a measure of a possible biologically adverse response. In case NP toxicity is investigated in a comprehensive study, however, involvement of in vivo experiments is crucial.

## Conclusion

Due to their interesting functional properties, numerous applications of NPs exist, e.g., plasmonic NPs [2,75], magnetic NPs [162–163] or fluorescent NPs [164]. For optimizing NP properties for biological applications, an understanding of their interaction with mammalian cells needs to be gained. However, the interaction of NPs with cells is complex due to the many different types of NPs, cells, and exposure scenarios being used within the field*.* Still, one may make an attempt to reduce details to very general statements, in order to highlight some essential elements, which was the motivation for this review.

Endocytosis is the common route of NP uptake. NPs which strongly interact with the cell plasma membrane are also internalized more efficiently. Hereby differences in uptake are not digital (i.e., "yes" or "no"), but rather are based on different concentration-dependent kinetics. After internalization NPs inside intracellular vesicles are in an environment (acidic pH, enzymes) completely different from that in the cytosol and the extracellular space, which can modify their properties. The translocation of the NPs from these vesicles to the cytosol is a current challenge, which is referred to as endosomal escape dilemma. Uptake studies best should involve a quantitative distribution analysis. While endocytotic uptake of NPs has been extensively investigated, the eventual loss of internalized NPs as a result of mitotic division, NP exocytosis, or NP transcytosis on the other hand has not been comprehensively studied yet. Cellular uptake studies of NPs require as much characterization of the NP material as currently possible. However, many physicochemical properties of NPs such as size, shape, charge, and colloidal stability are highly entangled, which complicates analysis. Analysis of physicochemical properties should be always performed in the incubation medium in which the uptake of NPs by cells is studied. The incubation medium can for example modify the colloidal stability of the NPs. Colloidal stability does not only interfere with size but also with other parameters such as shape. In general, small, elongated, and positively charged NPs are incorporated preferentially to big, flat, and uncharged NPs.
